# Tuberculosis and Hepatic Steatosis Are Prevalent Liver Pathology Findings among HIV-Infected Patients in South Africa

**DOI:** 10.1371/journal.pone.0117813

**Published:** 2015-02-10

**Authors:** Christopher J. Hoffmann, Jennifer D. Hoffmann, Caroline Kensler, Martin van der Watt, Tanvier Omar, Richard E. Chaisson, Neil A. Martinson, Ebrahim Variava

**Affiliations:** 1 Department of Medicine, Johns Hopkins University School of Medicine, Baltimore, United States of America; 2 University of Pittsburgh School of Medicine, Pittsburgh, United States of America; 3 Perinatal HIV Research Unit, University of the Witwatersrand, Johannesburg, South Africa; 4 Department of Anatomical Pathology, University of the Witwatersrand and National Health Laboratory Service, Johannesburg, South Africa; 5 Department of Medicine, Klerksdorp Tshepong Hospital Complex and University of the Witwatersrand, Klerksdorp, South Africa; University of Cincinnati College of Medicine, UNITED STATES

## Abstract

Liver disease epidemiology in sub-Saharan Africa has shifted as a result of HIV and the increased use of antiretroviral therapy leading to a need for updated data on common causes of liver disease. We retrospectively reviewed records from all hospitalized patients who had liver biopsy at a single hospital in South Africa from 2001 to 2009 and compared diagnosis by HIV status. During the period of study 262 patients had liver biopsy, 108 (41%) were HIV-infected, 25 (10%) were HIV-sero-negative, and 129 (49%) had unknown or unrecorded HIV status. Overall 81% of biopsies provided additional diagnostic data. Malignancy was the most common finding reported on 56 (21%) biopsies followed by granuloma or TB, hepatic steatosis, and fibrosis or cirrhosis. HIV-infected patients were more likely to have granulomas and steatosis. Half of patients with granulomas were already on TB treatment, suggesting paradoxical reactions or drug induced liver injury may have been important causes of liver inflammation among these patients. We note that TB, paradoxical reactions during TB treatment, possible drug induced liver injury, and hepatic steatosis are important causes of liver pathology among HIV-infected hospitalized patients with unclear etiology of liver disease after initial assessment. Among HIV sero-negative patients, malignancy was the major cause of liver disease. Our findings re-enforce the importance of TB as a diagnosis among HIV-infected individuals.

## Introduction

Biochemical abnormalities suggesting liver disease are common among patients [1], particularly hospitalized patients [2]. Liver abnormalities in transaminases, bilirubin, and alkaline phosphatase can be due to primary liver disease or may be a manifestation of systemic illness; determining the cause may guide therapy [2,3]. In settings with constrained clinical and diagnostic resources identification of etiology can be challenging. In many resource-limited settings a valuable tool for assessing liver disease, liver biopsy, is challenging or impossible to obtain. In settings where extensive investigations are not possible, understanding the local epidemiology of liver disease is essential for developing a differential diagnosis to guide decision making.

The emergence of HIV in Africa has led to an increase in opportunistic infections, particularly tuberculosis, and HIV-related malignancies, all of which may involve the liver [4–6]. In addition, medications used to treat these conditions, including antiretroviral therapy (ART), preventive therapies prescribed to people living with HIV (cotrimoxazole and isoniazid), and TB treatment have potentially hepatotoxic effects. Chronic viral hepatitis, especially hepatitis B virus, are also important causes of morbidity in Africa and are likely to have accelerated progression among HIV-co-infected individuals [7]. Insight into current causes of liver disease is important for clinicians working in sub-Saharan Africa. To characterize current epidemiology of liver disease we retrospectively reviewed clinical liver biopsy findings in HIV-infected, sero-negative, and unknown status individuals with liver disease of unclear etiology after clinical, laboratory, and radiographic assessments at a single center in South Africa.

## Methods

### Population

This research was conducted according to the principles expressed in the Declaration of Helsinki. This study was retrospective without interaction with patients, thus written informed consent was not obtained. All data abstraction forms were devoid of personal identifiers (all data were de-identified prior to data capture and data analysis). Approvals for the study were received from the Johns Hopkins University and the University of the Witwatersrand ethics committees.

Patients included in this study were adult patients (18 years and older) admitted to a single regional hospital in the North West Province of South Africa, between 2001 and 2009 who underwent a liver biopsy during their stay. This hospital draws patients from a catchment area with both a high prevalence of HIV (20% adult prevalence) and TB [8]. Patients who underwent liver biopsy were without a clear diagnosis after initial clinical evaluation, laboratory testing, and imaging. Individuals with symptoms and laboratory tests consistent with acute viral hepatitis, hepatocellular carcinoma, liver abscess, metastatic cancer, or who had rapid resolution of the hepatitis (within 2–5 days of admission) were generally not considered candidates for biopsy. A single internist evaluated all patients for biopsy and performed all liver biopsies during the study period. Biopsy results were obtained from the National Health Laboratory Service Anatomical Pathology Department. Hospital charts from the period of admission during which the biopsy was done were abstracted onto a standardized tool by trained staff.

### Analysis

We categorized histopathological results, using the dominant reported pattern, into the following five general groups: granuloma with or without acid fast bacilli, hepatic steatosis, cholangiopathy, fibrosis or cirrhosis, and malignancy. Granuloma and acid fast bacilli were combined in one group as this finding was nearly always either reported to be consistent with TB, or granulomas were reported to contain acid fast bacilli. We described the demographic data and results by HIV status, classified as known HIV-sero-negative, HIV-infected, and unknown HIV status. Included with cholangiopathic processes are processes of bile duct degeneration and vanishing bile duct syndromes; these syndromes fit the broader pathological description of ductopenic processes. Therefore we have used the term ductopenic to describe this subset of cholangiopathy. Chi-square, Wilcoxon rank sum, and Kruskal-Wallis tests were used to assess for differences with an alpha of <0.05. For patients with HIV, we have presented the range of CD4 count and biochemical test results for each diagnostic category along with Kruskal-Wallis testing for difference. We further provided an overall description of additional histopathologic findings, especially findings considered secondary during the histopathological review.

## Results

Between January 2001 and December 2009, 262 patients with liver disease of unclear etiology had a liver biopsy. Of these, 108 (41%) were HIV-infected, 25 (10%) were HIV-sero-negative, and 129 (49%) had unknown or unrecorded HIV status during the hospitalization (Table 1). HIV-infected patients were younger, with a median age of 34 years [interquartile range (IQR): 31, 45] compared to 55 years (IQR: 40, 64; Wilcoxon rank sum p<0.001) for those sero-negative for HIV. Overall, 95% were black, 1% were Asian, and 4% were white. HIV-infected patients were receiving ART (44%), cotrimoxazole (34%), and/or TB treatment (44%); 19% were receiving all three. Among HIV-sero-negative and HIV unknown status patients, 3 (13%) and 2 (2%), respectively, were receiving TB treatment at the time of biopsy. An alcohol history was recorded in the medical records of 35 patients; 20 reported no alcohol use, 3 reported moderate, and 12 reported heavy alcohol consumption. Prevalence of hepatic seromarkers in those tested was hepatitis A IgM positive among 0 of 81; hepatitis B surface antigen positive among 8 of 81 (10%); hepatitis C antibody positive among 4 of 80 (5%; no HCV RNA results were available), and *Schistosoma* antibody, positive among 0 of 44.

**Table 1:** Patient characteristics.

	HIV-infected	HIV-sero-negative	unknown HIV status
N	108	25	129
Sex			
Male	49 (45)	15 (60)	62 (48)
Female	59 (55)	10 (40)	67 (52)
Age	34 (31, 45)	55 (40, 64)	47 (34, 56)
Reported alcohol use (35 with recorded history)			
None	15 (65)	1 (20)	4 (57)
Moderate	1 (4)	0	2 (28)
Heavy	7 (30	4 (80)	1 (14)
HBsAg results			
Negative	49 (45)	10 (40)	13 (10)
Positive	8 (7)	1 (4)	1 (1)
Not tested	51 (47)	14 (56)	115 (89)
HCV antibody test result			
Negative	56 (52)	8 (32)	12 (9)
Positive	2 (2)	1 (4)	1 (1)
Not tested	50 (46)	16 (64)	116 (90)
Receiving TB treatment on admission	48 (44)	3 (13)	2 (2)
Receiving ART on admission	48 (44)	NA	NA
Receiving cotrimoxazole on admission	(34)	NA	NA
Histopathologic diagnoses			
Granuloma or TB	28 (26)	2 (8)	11 (8)
Cholangiopathy	12 (11)	3 (12)	6 (4)
Steatosis	23 (21)	3 (12)	10 (8)
Malignancy	13 (12)	6 (24)	37 (29)
Fibrosis	9 (8)	5 (20)	22 (17)
Non-diagnostic or inadequate	16 (15)	9 (36)	32 (25)

HBsAg: hepatitis B surface antigen

HCV: hepatitis C virus

Overall 81% of biopsies provided diagnostic data. Among HIV-infected patients, 15% were non-diagnostic compared with 8% of HIV sero-negative patients and 25% with unknown HIV status. Findings were as follows: granuloma (with or without identification of acid fast bacilli), 41 (16%); cholangiopathy, 21 (8%); steatosis, 36 (14%); malignancy, 56 (21%); fibrosis, 36 (14%); other, 21 (8%); a non-diagnostic pattern 45 (17%), and an inadequate specimen 6 (2.2%) (Table 1). Of the 36 with fibrosis, 12 (33%) had cirrhosis. Ten of the biopsies were consistent with drug induced liver injury; 8 of these were among HIV-infected patients.

Compared to HIV seronegative patients, those infected with HIV were more likely to have granulomas or definite TB; 28/108 (26%) patients with HIV compared to 2/25 (8%) HIV-seronegative patients and 11/129 (8.5%) with unknown HIV status; p = 0.001. Forty-nine percent of patients with granulomas were receiving TB treatment at the time of hospitalization (prior to biopsy), 19 of whom were HIV-infected, 1 was HIV-negative, and there were none with unknown HIV status. The majority with HIV were also receiving ART [15 of 28 (53%)]; the median CD4 count for those with HIV and granulomas was 135 cells/mm^3^ (IQR: 69, 226).

Compared to HIV sero-negative patients, those infected with HIV were more likely to have steatosis [30/108 (28%) versus 3/25 (12%), p = 0.001]. Female sex was also associated with steatosis (34% of all women versus 17% of men, p = 0.02). Twelve of the 30 with HIV were on ART at the time of admission, all were either receiving stavudine (5) or the ART regimen was not recorded (7) but was likely to have been stavudine given the guidelines in use at that time. The median CD4 count among HIV-infected patients with steatosis was 176 cells/mm^3^ (IQR: 91, 253). We did not identify any other risk factors for steatosis.

HIV-infected patients were also more likely to have cholangiopathy compared to HIV sero-negative patients [19/108 (18%) compared to 3/25 (12%), p = 0.006]. Five HIV-infected patients had the specific diagnosis of HIV-associated cholangiopathy based on histopathology (others may also have had HIV-associated cholangiopathy as the diagnosis generally uses clinical and histopathological data). In addition, among HIV infected adults, ductopenic histopathology was associated with cotrimoxazole use (35% receiving cotrimoxazole versus 8% not receiving cotrimoxazole, p = 0.001) and TB treatment (27% receiving TB medications versus 10% not receiving TB treatment, p = 0.02). Of note, 58% of patients receiving TB treatment were also receiving cotrimoxazole. The median CD4 count among HIV-infected patients with cholangiopathy was 105 cells/mm^3^ (IQR: 60, 494). There was no association with those receiving ART and cholangiopathy.

Fibrosis or cirrhosis was not associated with HIV serostatus, but was increased with female sex (p = 0.02) and older age trended toward association (p = 0.06). The median CD4 count among HIV-infected patients with fibrosis or cirrhosis was 365 cells/mm^3^ (306, 513). When excluding fibrosis and limiting analysis to cirrhosis, older age was associated with cirrhosis versus not having cirrhosis (p = 0.004) while female sex lost association.

Malignancies were diagnosed in 21% of patients; men were more likely to have a biopsy demonstrating malignancy than women [15/60 (25%) men compared to 6/73 (8%) women, p = 0.008]. Most malignancies were found among individuals who were sero-negative for HIV or had unknown HIV status. The exception was lymphoma: 8 of the 11 with lymphoma were HIV-infected, representing 62% of all malignancies diagnosed among HIV-infected individuals (the other 3 lymphoma cases were among the unknown HIV status group). The types of malignancy were as follows: metastatic adenocarcinoma, 13 (39%); hepatocellular carcinoma, 11 (20%); lymphoma, 11 (20%); and other, 12 (21%). Specific testing for viral hepatitis was not completed on any of the patients diagnosed with hepatocellular carcinoma.

A number of additional findings were reported. For example, three patients had biopsy findings consistent with chronic hepatitis B, one with chronic hepatitis C, and one with *Schistosoma* infection. Specific serology was available for only one of the patients, one with biopsy findings consistent with chronic hepatitis B and serology positive for HBsAg. Three additional patients had a secondary finding of iron overload, two of whom had primary diagnoses of granulomas consistent with TB and one had a primary diagnosis of cirrhosis without a clear etiology.

Among HIV-infected patients we additionally compared biochemical test results by diagnostic category (Table 2). Patients with cholangiopathy had the lowest median CD4 count (although p>0.05) and highest median alkaline phosphatase [median: 462 (IQR: 328, 1757)]. AST was highest among patients with granulomas and steatosis [105 U/L (IQR: 72, 260) and 112 U/L (101, 122), respectively] but those differences were not statistically significant. Median values of other biochemical markers varied less by pathology.

**Table 2:** Primary histopathology and laboratory findings for HIV-infected patients.

	Granulomatous disease / TB	Cholangiopathy	Steatosis	Malignancy*	Fibrosis/cirrhosis	p
	Median (IQR)	Median (IQR)	Median (IQR)	Median (IQR)	Median (IQR)	
n (%)	28 (26)	11 (10)	23 (21)	11 (10)	7 (6)	
CD4 count (cells/mm^3^)	135 (69, 226)	105 (60, 494)	176 (91, 253)	225 (89, 362)	365 (306, 513)	0.5
ALT (U/L)	57 (45, 112)	31 (20, 63)	50 (41, 76)	194	63 (43, 113)	0.5
AST (U/L)	105 (72, 260)	47 (10, 59)	112 (101, 122)	271	98 (52, 155)	0.4
Alkaline phosphatase (U/L)	382 (219, 713)	462 (328, 1757)	275 (260, 359)	1913	200 (187, 307)	0.03
Total bilirubin (μmol/L)	28 (13, 40)	11 (5, 20)	18 (12, 33)	96	24 (19,105)	0.5

*Patients with malignancy lacked most laboratory data

## Discussion

There are limited data on histopathologic findings among hospitalized individuals with liver disease from a high HIV prevalence African setting. We believe that our study adds further insight regarding common patterns of clinically undiagnosed liver disease among patients in this setting. Our findings highlight the role tuberculosis and drug induced liver injury in contributing to liver derangements. Cotrimoxazole was most clearly associated with drug induced liver injury as it was associated with a ductopenic process, a previously observed association [9,10]. We also note a high proportion of HIV-infected individuals had hepatic steatosis.

Increased granulomatous liver disease among HIV-infected individuals is consistent with the known association between TB and HIV. However, the relative proportion of liver disease of initial unclear etiology caused by either TB disease (previously undiagnosed) or anti-TB therapy or paradoxical reaction to TB has not been previously described [11,12]. A drug injury may warrant a modification in the TB treatment regimen, while a paradoxical reaction should not prompt a change in regimen [13]. Among patients already on stable ART and TB treatment, we suspect that drug injury contributed to liver inflammation and was interpreted as such by the pathologist among 10 patients. Notably, we cannot exclude the possibility of a paradoxical reaction misinterpreted as a drug reaction. Drug induced liver injury is commonly reported among patients with HIV, especially those receiving ART [14–18]. These studies reporting drug induced liver injury among patients receiving ART are generally based on serum laboratory markers and rarely include histopathological findings or data to exclude other processes. Depending on the local prevalence of TB and other illnesses and the ART agents used, it is plausible that the reported incidence of hepatotoxicity from such studies also includes non-drug related liver disease [19].

Hepatic steatosis is a complication of some antiretroviral therapy, especially stavudine, an agent that was widely used in South Africa during the time-frame of this study [20]. In addition to medications, steatosis is associated with alcohol use and obesity, both of which are highly prevalent in South Africa [21–23]. However there is no reason to believe that obesity or alcohol use are significantly increased among the HIV-infected individuals compared to HIV-seronegative or unknown status patients in this study. Thus the steatosis we found is likely due to HIV infection, its treatment, or both. Hepatic steatosis can progress to liver fibrosis and a decline in liver function and is considered an important cause of chronic liver disease in high-income countries [21]. With increasing survival among people living with HIV in low and middle income settings it may also become an important cause of morbidity in such settings.

This study has the strength of data coming from a routine practice with a single physician selecting patients for biopsy and performing the biopsies. Clear limitations include the lack of representative sampling and missing data. HIV status was unknown for many patients because, during part of the study period, HIV testing was predominantly available in the outpatient setting. Other data were variably missing, for example most HIV-infected patients with malignancy did not have results from biochemical testing and hepatitis B antigen testing was completed for less than half of the patients the study. In addition, this study reflects only the subset of patients with liver disease of unclear etiology after an initial evaluation who then went on to have a liver biopsy. This is an important limitation because patients with laboratory diagnosed hepatitis B or liver masses associated with a markedly elevated alpha-fetoprotein were generally unlikely to get a biopsy. We highlight that chronic viral hepatitis, especially chronic hepatitis B, is an important cause of liver disease, both chronic and acute, in much of Africa [7]. Alcoholic hepatitis and alcohol related chronic liver disease are additional important causes of liver disease in South Africa [24]. Because of the nature of this study, we would expect both hepatitis B-related and alcohol-related liver disease to be underrepresented among patients who had biopsies for the purposes of diagnosis. Finally, we included HIV-sero-negative and unknown HIV status patients in order to provide a point of comparison. It is important to note that we had a small sample of HIV-negative patients and that those with unknown HIV status represent a mix of HIV-infected and sero-negative patients.

Liver disease is an important cause of morbidity in resource limited settings [25,26]. In settings similar to the one studied, with a high prevalence of HIV and TB, we believe our results may help to refine a differential diagnosis. Furthermore the findings highlight the importance of undiagnosed TB and TB medications or paradoxical reaction as a cause of liver disease.

## Supporting Information

S1 Dataset. (CSV)
